# Inhibitory actions of selected natural substances on formation of advanced glycation endproducts and advanced oxidation protein products

**DOI:** 10.1186/s12906-016-1353-0

**Published:** 2016-09-29

**Authors:** Ewa Grzebyk, Agnieszka Piwowar

**Affiliations:** 1 Department of Pharmaceutical Biochemistry, Wroclaw Medical University, Faculty of Pharmacy, Borowska 211A St., 50-556 Wroclaw, Poland; 2 Department of Toxicology, Wroclaw Medical University, Faculty of Pharmacy, Borowska 211 St., 50-556 Wroclaw, Poland

**Keywords:** Glycation, Oxidation, Advanced Glycation Endproducts (AGE), Advanced Oxidation Protein Products (AOPP), High- and Low-Molecular-Weight fractions (HMW and LMW), Vitamin C, Green tea, Quercetin, Padma 28, Padma Circosan

## Abstract

**Background:**

Advanced glycation endproducts (AGE) and advanced oxidation protein products (AOPP) arise as a result of excessive glycation and oxidation processes of proteins in hyperglycemia and oxidative stress conditions respectively, both in vivo and in vitro. In vivo these processes are especially intensified in patients with diabetes, and the adverse effects of AGE and AOPP are particularly unfavorable for the pathogenesis and aggravate the biochemical disturbances and clinical complications of diabetes. Total AGE and AOPP (T-AGE and T-AOPP) are heterogeneous groups of compounds, and they can be divided into two main fractions: high- and low-molecular-weight, i.e. HMW-AGE and HMW-AOPP as well as LMW-AGE and LMW-AOPP. Therefore it is important to find natural substances that will prevent formation of total AGE and AOPP and their high- and low-molecular-weight fractions and thereby reduce their adverse effects on tissues and organs.

**Method:**

Selected natural substances and dietary supplements such as vitamin C, aminoguanidine, quercetin and green tea as well as the multicompound formulations Padma Circosan and Padma 28 were tested in an in vitro model using bovine serum albumin (BSA). Fluorescence of T-, HMW- and LMW-AGE and concentration of T-, HMW- and LMW-AOPP were measured after incubation with these substances.

**Results:**

In the examined concentrations quercetin showed the greatest degree of inhibition for T-AGE (60.5 %) as well as for HMW-AGE (79.5 %), while in the case of LMW-AGE the greatest degree of glycation inhibition was shown by Padma Circosan (74.9 %). T-AOPP and HMW-AOPP were best inhibited by vitamin C (87.3 and 89.1 % respectively). The results obtained for LMW-AOPP are atypical, but the lowest concentration was observed in a sample with Padma 28.

**Conclusion:**

The results show that all tested natural compounds have inhibitory activity towards the formation of total and low- and high-molecular-weight forms of AGE and AOPP in vitro. That suggest a possible role in the prevention of diabetic complications, especially the multiherbal compound Padma preparations, which are especially effective in lowering the most dangerous, i.e. LMW fractions.

## Background

The process of nonenzymatic glycation in in vivo conditions occurs in almost all tissues and organs of the body, and physiologically is associated with aging. However, its intensity is significantly increased in diabetes, which is characterized by a state of chronic hyperglycemia. In these patients oxidative stress is also very often increased, leading to oxidative damage of macromolecules and tissues [1, 2]. Nonenzymatic glycation and oxidation concerns proteins, lipids and nucleic acids, which cause a series of adverse changes in their structure and function. The multi-stage conversion, initiated by a glucose connection to free amino groups of proteins, leading to the creation of glycation products, is commonly known as the Maillard reaction. The last stage of this reaction is the irreversible formation of advanced glycation endproducts (AGE). The amount of these compounds reflects the intensity of protein glycation and its severity. Similarly, as a result of multi-stage transformations of proteins induced by oxygen and chlorine free radicals, advanced oxidation protein products (AOPP) are formed. The main sources of oxidative compounds are neutrophils, which are especially stimulated in hyperglycemic conditions. AGE and AOPP are also known as endogenous toxins, and especially AOPP were defined as uremic toxins in patients with end stage renal disease [2–5]. Although the exact structure and properties of AGE and AOPP are still not well recognized in spite of having been intensively studied, strong similarity of these compounds is indicated. In particular, their participation in the pathogenesis and development of vascular late diabetic complications, including micro- and macroangiopathies, is emphasized, and their role in diabetic nephropathy is especially underlined. They are often the cause of rapidly deteriorating health of diabetic patients and can lead to significant shortening of their lifespan. In addition, AGE and AOPP enhance the mechanisms related to inflammation that lead to the respiratory burst of phagocytic cells, which secondarily intensifies their increased formation [1, 2, 6–8].

AGE are a heterogeneous group of compounds, defined as total AGE (T-AGE), mostly characterized by a specific fluorescence and specific properties. They occur in two main forms: high-molecular-weight AGE (HMW-AGE, protein aggregates and their fragments, size up to 650 kDa) and low-molecular-weight AGE (LMW-AGE, molecular weight lower than 12 kDa) fractions [3, 6, 9]. Similarly, total AOPP (T-AOPP) comprise a large group of compounds with not fully defined structure, which also occur as high-molecular-weight AOPP (HMW-AOPP, molecular weight of up to 600 kDa) and low-molecular-weight AOPP (LMW-AOPP, lower than 12 kDa) [5, 10]. They are currently being intensively investigated because of their role in tissue and organ injury. Excessive AGE and AOPP formation and accumulation is particularly implicated in kidney dysfunction, and very high levels were found in patients with uremia and end-stage renal diseases [10–13]. In our previous studies involving patients with type 2 diabetes, we revealed a significant relationship of both AGE and AOPP with development of diabetic angiopathies [14]. Moreover, some studies indicate that LMW-AGE and LMW-AOPP are more toxic than HMW species [12, 13]. It is therefore vitally important to find a substance that can inhibit both total as well as high- and low-molecular-weight AGE and AOPP formation [3]. Besides the endogenous formation, AGE are also taken up externally with food, which augments the unfavorable effect on the organism [6, 15, 16].

Our previous studies on the in vitro model allowed us to find few substances of natural origin, which showed a great ability to inhibit unfavorable processes of glycation and oxidation (more than 50 % braking efficiency of each of these processes). These substances are included in the supplements commonly used in drugs and medicines available without a prescription and are frequently bought and used by people particularly vulnerable to oxidative stress and increased glycation. As a result of our previous studies of the best inhibiting processes of glycation and oxidation compounds we include vitamin C, green tea, aminoguanidine, quercetin and preparations Padma 28 and Padma Circosan [17, 18]. Due to the fact, that LMW fractions of AGE and AOPP are thought to be even more dangerous than the HMW fractions [14] the natural substances mentioned above were analysed regarding there inhibiting effects specifically on these two main forms of AGE and AOPP.

## Methods

### Test substances and materials

Bovine serum albumin (BSA) was used as a model protein in a concentration of 40 mg/mL (corresponding to the physiological albumin concentrations in human blood). The glycation and oxidation processes were carried out using glucose and chloramine T solution in PBS (500 mg/mL and 20 mM respectively) as activators. Solutions of selected natural origin substances with known anti-oxidative and/or antiglycative effects were used: aminoguanidine (A) and quercetin (Q) in concentrations of 50 mM, vitamin C (ascorbic acid, VC) and green tea (GT) in a concentration of 10 mM, Padma Circosan (PC) and Padma 28 (P28) in a concentration of 2.5 %. Padma Circosan and Padma 28 are multiherbal formulations from 21 and 22 powdered herbal drugs respectively. They are identical except that Padma 28 additionally contains aconite tuber (1 mg/403 mg). The full composition has been published elsewhere [19]. All concentrations were chosen based on the literature and our own previous experiments (data not shown). As a negative control C(−) native BSA solution was used, while as a positive control C(+) BSA solution was incubated with glucose for glycation or with chloramine T for oxidation processes. Each experiment was performed three times in triplicate.

Used in the study vitamin C, quercetin and aminoguanidine were the purest available from Sigma Aldrich (US) substances (based on our previous studies [17, 18] we decided to use these natural origin substances). Padma 28 and Padma Basic preparations were obtained from the manufacturer (Padma Inc., CH), while green tea was from a dietary supplement from the company Aboca (IT) (the composition contains green tea leaves and pure green tea extract; preparation was measured on the epigallocatechin gallate concentration). All other reagents used in the study were from Sigma Aldrich (US).

### Glycation

To measure AGE formation, samples were incubated for 6 weeks at 37 °C in darkness, and then dialyzed (48 h in PBS, pH 7.4, with one change of dialysis solution). The characteristic fluorescence of total AGE as well as its high- and low-molecular-weight fractions – i.e. HMW-AGE and LMW-AGE (after their separation according to the method described by Munch et al. [20]) – was measured. A Perkin Elmer spectrofluorometer was used and 370 and 440 nm were applied as excitation and emission wavelength, respectively. The results were converted according to the following formula: AGE [AFU] = (F × D) × 1000/V, where: F = value of the measured fluorescence, D = sample dilution (500 times – T-AGE, 20 times – HMW-AGE and LMW-AGE) and V = volume of used sample (20 μL – T-AGE, 100 μL – HMW-AGE and LMW-AGE). The results are presented in AFU (arbitrary fluorescence units) as the mean of three measurements.

### Oxidation

The concentrations of total AOPP as well as its high- and low-molecular-weight fractions – i.e. HMW-AOPP and LMW-AOPP – were measured according to the method described by Vitko-Sarsat et al. [5]. Separation of LMW- and HMW-AOPP was performed in similar conditions as in the case of AGE separation [20]. To measure AOPP formation the samples were incubated for 60 min at 37 °C in darkness, and then dialyzed (48 h in PBS, pH 7.4, with one change of dialysis solution). The characteristic colour at 340 nm was measured in a Stat Fax 2100 spectrophotometer. The standard curve (made in chloramine T with concentration from 0 to 100 μM) was used to calculate the AOPP concentration and expressed in μM converted according to the formula: AOPP [μM] = 1 / B × A × D × 1000, where A = absorbance of measured sample, D = dilution (10×), B = slope of the calibration curve (B = 0.0141). The results are presented as the mean of three measurements.

### Electrophoresis

To confirm the accuracy of the applied procedure of AGE and AOPP formation and their proper separation into high- and low-molecular-weight fractions of AGE and AOPP, electrophoresis was performed according to the method described by Laemmli et al. [21]. A polyacrylamide gel with Coomassie Brilliant Blue G-250 reagent staining was used.

### Statistical analysis

Statistical analysis was performed using Statistica 12.5 PL. The average and standard deviation (SD) were calculated. Next, we performed an analysis of the results for examined samples and a negative control as compared to a positive control using Student's t test. A *P* value less than 0.05 was adopted as statistically significant.

## Results

The levels of total AGE and total AOPP as well as their HMW and LMW fractions in positive and negative controls as well as in samples incubated with selected natural substances are presented in Table 1. Bold font indicates the lowest fluorescence/concentration of the measured parameter, reflecting the strongest inhibitory effect of the examined substances toward AGE and AOPP formation in in vitro conditions. All tested samples and negative controls were statistically different compared to C (+) (*p* <0.001).

**Table 1:** Results of fluorescence of AGE (T, HMW and LMW) and concentrations of AOPP (T, HMW)

	T-AGE [AFUx10^4^]	HMW-AGE [AFUx10^3^]	LMW-AGE [AFUx10^3^]	T-AOPP [μMx10^3^]	HMW-AOPP [μM]	LMW-AOPP [μM]
C(−)	3.6	3.0	1.1	5.0	87.4	36.9
C(+)	17.0	24.7	5.5	152.3	893.4	11.7
A	7.7	10.0	1.5	55.6	190.2	18.4
VC	7.4	11.4	1.8	**19.4**	**97.7**	n. a.
Q	**6.7**	**5.1**	1.9	111.5	555.3	16.3
GT	8.1	11.1	1.8	46.8	375.4	14.9
P28	7.5	11.3	1.6	53.7	447.3	**12.8**
PC	8.1	12.1	**1.4**	47.9	339.4	14.2

C(−) – negative control, C(+) – positive control, samples incubated with: *A* aminoguanidine, *VC* vitamin C, *Q* quercetin, *GT* green tea, *P28* Padma 28, *PC* Padma Circosan

*n. a*. not applicable due to interference of the test substance with reagents used for isolation of LMW-AOPP

**Fig. 1:**
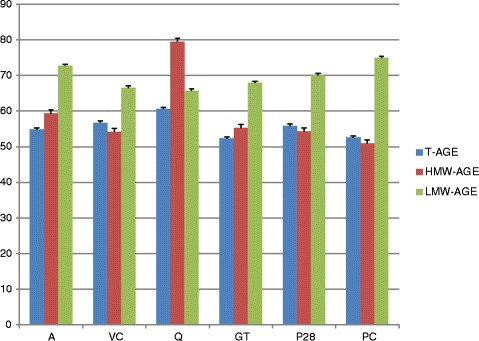
Percentage inhibition of formation of all forms of AGE. Samples incubated with: A – aminoguanidine, VC – vitamin C, Q – quercetin, GT – green tea, P28 – Padma 28, PC – Padma Circosan. SD values for each sample are marked on the corresponding bars

**Fig. 2:**
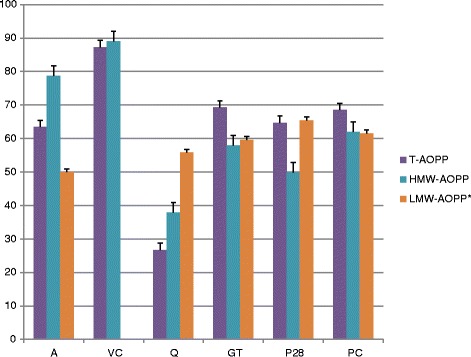
Percentage inhibition of formation of all forms of AOPP. Samples incubated with: A – aminoguanidine, VC – vitamin C, Q – quercetin, GT – green tea, P28 – Padma 28, PC – Padma Circosan. SD values for each sample are marked on the corresponding bars. * Due to an atypically lower value for C+ compared to C- the inhibition of LMW-AOPP was calculated as % against the C(−) value as 100 %

For each tested compound the percentage inhibition of glycation and oxidation processes compared to C(+) was calculated (Figs. 1 and 2). In the case of LMW-AOPP, because of the obtained atypical results, the percentage inhibition was compared to C(−). For each sample was SD calculated and also marked on Figs. 1 and 2.

The effectiveness of the procedure of glycation and oxidation of BSA was confirmed by the differences in the concentration or fluorescence of all forms of AGE and AOPP between positive and negative controls except in the case of LMW-AOPP (where because of the atypical results the inhibition was calculated as % against the C- value as 100 %). The largest difference was observed for T-AOPP, where values obtained in the positive control were about 30.5 times higher compared to C(−). Only in the case of LMW-AOPP were the obtained results atypical – the negative control was higher than in the positive one. As shown in Table 1 and Figs. 1 and 2, formation of T-AGE and T-AOPP was inhibited by all examined substances – in the case of AGE to the extent of more than 50 % and in the case of AOPP to the extent of more than 60 % (except quercetin). The best inhibitory effects of T-AGE and T-AOPP formation were achieved by quercetin (60.5 %) and vitamin C (87.3 %) respectively. In the case of the other examined substances T-AGE formation was inhibited by 56.7, 55.9, 54.9, 52.6 and 52.3 % in samples incubated with vitamin C, Padma 28, aminoguanidine, Padma Circosan and green tea respectively. T-AOPP formation was inhibited by 69.3 and 68.5 % in samples incubated with green tea and Padma Circosan, by 64.7 % with Padma 28, by 63.5 % with aminoguanidine, and by 26.8 % in samples incubated with quercetin.

The formation of HMW fractions was also the most inhibited by the same substances as T-AGE and T-AOPP respectively. HMW-AGE formation was the most strongly inhibited (the lowest HMW-AGE fluorescence) by quercetin (79.5) and HMW-AOPP was best inhibited by vitamin C (89.1 %). For all examined substances the inhibitory effect on HMW-AGE formation was higher than 50 %, and the HMW-AOPP formation inhibition was higher than 60 % in all samples except quercetin. HMW-AGE formation was 59.4 % inhibited by aminoguanidine, 55.3 % by green tea, 54.3 % and 54.1 % by Padma 28 and vitamin C respectively, and 50.9 % by Padma Circosan. In the case of HMW-AOPP formation, aminoguanidine inhibited the oxidation process by 78.7 %, Padma Circosan by 62.0 %, green tea by 58.0 %, Padma 28 by 49.9 %, and quercetin only by 37.8 %.

The results obtained for the LMW-AGE revealed the best inhibitory effect for samples incubated with Padma Circosan. The level of LMW-AGE in these samples is 74.9 % lower than in C(+). The LMW-AGE formation was inhibited by 72.7, 70.0, 67.9, 66.5 and by 65.8 % in samples incubated with aminoguanidine, Padma 28, green tea, vitamin C and quercetin, respectively. The inhibition of LMW-AGE formation was higher than 65 % in all examined samples. In LMW-AOPP obtained values differed by 50.0 to 65.4 % in examined substances compared to the negative control. LMW-AOPP concentration in the sample with Padma 28 was 65.4 % higher than in the negative control, and results obtained for remaining examined samples were 61.5, 59.6, 55.8 and 50.0 % higher in samples incubated with Padma Circosan, green tea, quercetin and aminoguanidine respectively. The LMW-AOPP formation was modified by more than 50.0 % in all examined samples. The result obtained for vitamin C cannot be analyzed due to interference of the test substance with reagents used for the isolation of LMW-AOPP (interference was observed even in an aqueous solution containing only vitamin C).

**Fig. 3:**
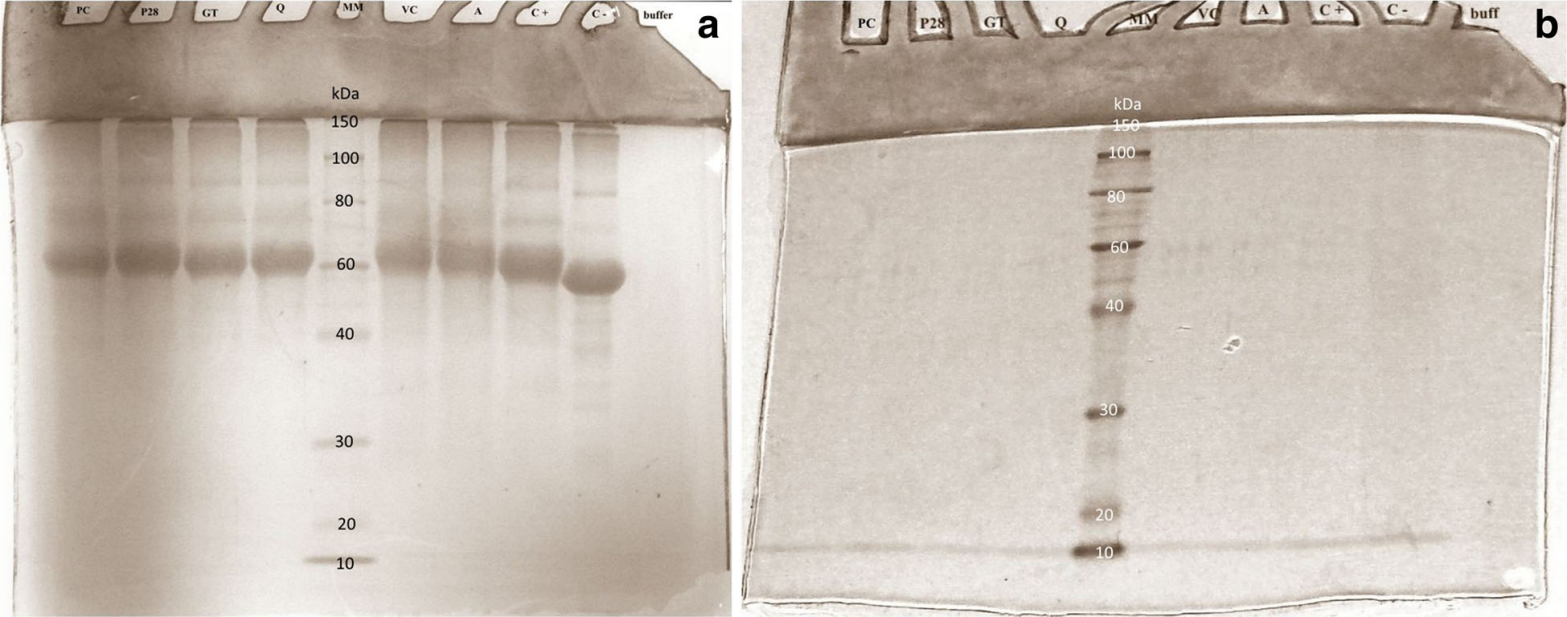
Check of effectiveness of the separation of HMW and LMW AGE: electrophoresis **a** HMW-AGE, **b** LMW-AGE. MM – mass marker, buffer – PBS, C - - negative control, C + −positive control, A –aminoguanidine, VC – vitamin C, Q – quercetin, GT – green tea, P28 – Padma 28, PC – Padma Circosan

**Fig. 4:**
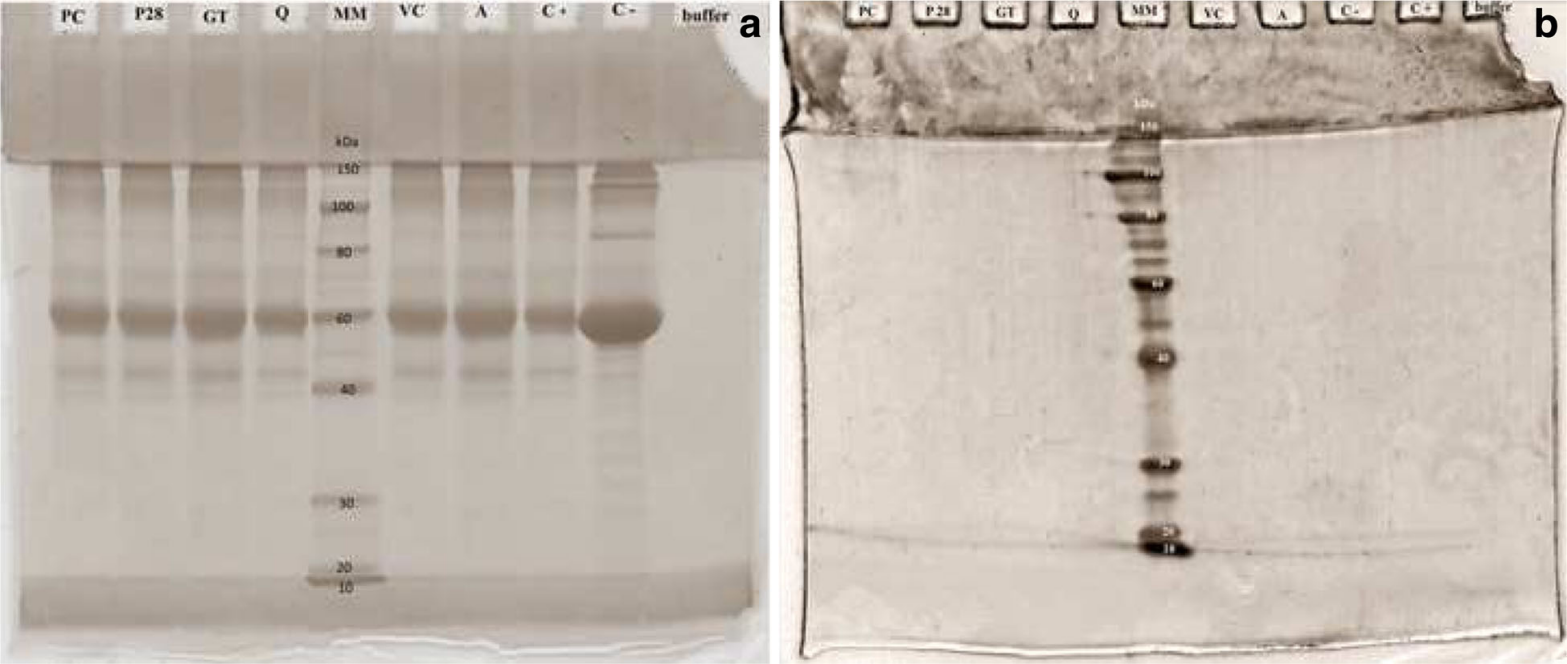
Check of effectiveness of the separation of HMW and LMW AOPP: electrophoresis **a** HMW-AOPP, **b** LMW-AOPP. MM – mass marker, buffer – PBS, C - - negative control, C + −positive control, A –aminoguanidine, VC – vitamin C, Q – quercetin, GT – green tea, P28 – Padma 28, PC – Padma Circosan

To check the effectiveness of the oxidation and glycation process and of the separation of the HMW and LMW fractions the samples were analyzed by gel electrophoresis. HMW fractions of AGE and AOPP are present in molecular weights of 40 kDa or more (because of the large mass of the aggregates, part of the protein did not enter the separating gel) in Figs. 3a and 4a. In Figs. 3b and 4b, a single band 1of low-molecular-weight protein can be seen at the end of the gel, corresponding to the size of 10 kDa or less. The electrophoresis results confirm the correctness of the separations of HMW and LMW fractions.

## Discussion

Non-enzymatic glycation and oxidation of macromolecules, particularly proteins, are strongly associated with hyperglycemia and oxidative stress conditions, which are especially increased in diabetes. The results of these processes are advanced glycation endproducts and advanced oxidation protein products. Nonenzymatic modifications alter the structure, function and properties of the proteins. AGE and AOPP are more resistant to proteolysis and are also more reactive, which leads to an increase in the synthesis of free radicals, reactive oxygen, nitrogen and chlorine species as well as protein radicals. At the same time, these macromolecules are more susceptible to subsequent oxidative modification themselves. This leads to the creation of vicious circle of disturbances that is particularly disadvantageous in patients with diabetes due to the role of AGE and AOPP in the pathogenesis and development of vascular late diabetic complications [1, 6–8]. In vivo high-molecular-weight fractions of these compounds are created by the crosslink reactions and connections of these glycated or oxidized proteins with other proteins and macromolecules (native and modified). On the other hand, low-molecular-weight fractions are mainly formed as a result of incomplete proteolysis of total AGE or AOPP or as products of incomplete glycation (also called “free AGE”) or oxidation. They also can be Maillard reaction products (early or middle glycation products), and they constitute a low percentage (10 % of all AGE) in the human blood. All fractions of AGE and AOPP are regarded as toxic agents for the organism, especially their low-molecular-weight fraction [3, 22]. They may accumulate in tissues and organs and activate RAGE receptors, which subsequently induce intracellular signaling pathways and start an inflammatory cascade. Thus AGE and AOPP may provoke chronic inflammation, disturb organ function and cause e.g. diabetic nephropathy or retinopathy development, diabetic atherosclerosis and cardiovascular disease [2, 5, 16, 23]. That is why it is so important to identify substances that inhibit the formation of these modified macromolecules, especially in view of their nephrotoxicity. This is the goal of recent intensive research efforts [2, 4, 9, 12]. We have evaluated the inhibitory effects of selected natural compounds, such as aminoguanidine, vitamin C, quercetin, green tea and polyherbal formulations such as Padma Circosan and Padma 28 on the formation of total AGE and AOPP as well as on their HMW and LMW fractions. The effectiveness of the experimental oxidation and glycation procedures was confirmed by the negative and positive controls. In the LMW-AOPP fraction the results for the controls were atypical – the positive control resulted in lower LMW-AOPP levels than the negative control. This suggests that while spontaneous oxidation results in increased formation of LMW-AOPP, in the experimental conditions the oxidized proteins show a tendency to aggregate rather than decay. Received by us results were repeatable in each of the independently performed series of experiments, so the authors decided to analyze and present the results in the manuscript. Therefore in the case of LMW-AOPP the results of the tested substances were compared to the negative control and not to the positive control.

The efficacy of the separation method of the HMW and LMW fractions of both AGE and AOPP was confirmed by electrophoresis – in the HMW fractions there are no proteins smaller than 40 kDa, and in the LMW fractions there are no proteins larger than 10 kDa. The electrophoretic images were comparable to the ones obtained by Vitko-Sarsat et al. [5].

Among the tested substances only aminoguanidine has well-known glycation-inhibiting properties. The other substances are known more for their antioxidant properties (e.g. vitamin C, green tea) [4, 24, 25]. The polyherbal formulations Padma 28 and Padma Circosan have known inhibitory effects on total AGE and total AOPP formation, as we have shown previously [26]. The ability to inhibit the total as well as the high- and low-molecular-weight fractions of AGE and AOPP has to our knowledge not yet been examined. The data available in the scientific literature indicate a possible inhibitory effect only on the total AGE [4, 7], or AOPP formation was investigated in general [2, 5, 17, 18, 26]. Currently there are no scientific studies that assess the ability of the compounds examined by us to inhibit or lower the HMW and LMW fractions of AGE and AOPP. In our previous investigations [17] we observed a strong inhibitory effect of both Padma preparations on formation of total AGE and AOPP in in vitro conditions, but there is no information about their effects on the high- and low-molecular-weight fractions of AGE and AOPP. The present study revealed that all of the tested substances show the effects on glycative and oxidative modification of proteins – in the experimental conditions they reduced the levels of all examined compounds. In the LMW-AOPP fractions all samples showed higher values than the positive control, and because of these atypical results the reduced LMW-AOPP levels were compared to the negative control.

The inhibitory effect of aminoguanidine on glycation is well described in the scientific literature and depends on the concentration of reagents, the incubation time, the temperature and the type of the glycation agent. In in vitro studies aminoguanidine inhibited the glycation process to a high degree. In some experiments aminoguanidine in a concentration of 10 mM inhibited the T-AGE formation by 75.4 and 64.8 % after 15 and 30 days of incubation respectively [27] or by 75 % in a concentration of 10 mM [28]. Hou et al. [29] observed almost complete inhibition of the glycation by aminoguanidine in a concentration of 100 mM. Furthermore, Gutierrez [30] demonstrated the ability of aminoguanidine to inhibit the glycation process by 58.3 % in in vitro conditions and confirmed this result in an in vivo study involving rats. Other researchers also demonstrated an antioxidative effect of aminoguanidine in rat models. These studies have shown its ability to lower the activity of nitric oxide and glutathione, and to lower the concentration of carbonyl groups, leading to reduced glycation [31, 32]. To our knowledge, up to now there has been no research on the effect of aminoguanidine on the formation of AOPP. Our experiments show that aminoguanidine, in a concentration of 50 mM, has a strong inhibitory effect (54.9 %) on the formation of T-AGE as well as on T-AOPP formation (63.5 %). Aminoguanidine also strongly inhibited formation of the HMW and LMW fractions of AGE and of HMW-AOPP and LMW-AOPP by 50–79 %.

As mentioned above, vitamin C is a substance with well-known antioxidant properties. Jing et al. [33] observed an antioxidant effect of vitamin C in a concentration of 0.3 mg/mL and inhibition of synthetic DPPH by 86 %. Also, Shah et al. [34] found that vitamin C has the ability to scavenge free radicals and lower their concentration by 84.6–98 % dose-dependently at concentrations from 20 to 100 μg/mL. Furthermore, Zhang et al. [35] reported that vitamin C in concentrations ranging from 6.25 to 200 mg/mL inhibits synthetic DPPH radicals and ABTS dose-dependently by 45–85 % and 49–95 % respectively. However, to our knowledge there are no studies on the action of vitamin C on AOPP formation or on the glycation process in in vivo studies. Tarwadi and Agte [36] demonstrated an inhibitory effect on glycation by about 20 %. The authors suggest that the effect of vitamin C involves reduction of oxidative stress, which may lead to a reduction of the escalating mechanisms between oxidation and glycation. Similarly, Lavelli et al. [37] also supported the ability of vitamin C to inhibit glycation, but stressed that its action still needs to be confirmed. In contrast, Vinson and Howard [7] demonstrated that vitamin C at a concentration of 20 mM inhibits the process of glycation and AGE formation by about 73 %. Our data confirm a strong inhibitory effect of vitamin C in a concentration of 10 mM not only on the oxidation but also on the glycation process, lowering T-AOPP and T-AGE formation by 87.3 and 56.7 % respectively. Vitamin C also showed strong inhibition of HMW- and LMW-AGE and of HMW-AOPP formation (54.1 to 89.1 % inhibition), and in the case of T-AOPP and HMW-AOPP it even showed the best inhibitory effect of all examined substances.

Quercetin, like vitamin C, is known as an antioxidant and, as some authors suggest, has a comparable antioxidative capacity. In concentrations of 6.25 to 200 mg/mL it inhibited synthetic DPPH dose-dependently by 50–95 % [35]. High antioxidant abilities of quercetin were also confirmed at lower concentrations ranging from 20 to 100 μg/mL, its inhibition of synthesis of DPPH radicals ranging from 95 to 98 % [34]. Inhibitory action of this radical comparable to that of vitamin C was demonstrated by Djouossi et al. [38]. However, to our knowledge, there have been no studies on the effect of quercetin on AOPP formation. Some researchers have reported an ability of quercetin to inhibit the glycation process in concentrations ranging from 50 to 200 μg/mL [39] and to inhibit AGE formation by 76–99 %, whereas other researchers demonstrated, at a concentration of 100 μg/mL, a capacity for glycation inhibition of only around 33 % [40]. In our experiment, quercetin showed the strongest inhibitory action on total AGE formation by 60.5 % in a concentration of 50 mM. However, the inhibitory effect on T-AOPP formation was the weakest of the examined substances, at 26.8 %. HMW and LMW fractions of AGE and HMW-AOPP and LMW-AOPP were inhibited by quercetin to a degree of 37.8 to 79.5 %, and it showed the strongest inhibitory effect on HMW-AGE formation.

Green tea is often described as a highly antioxidant beverage, and its oxidation inhibiting action has been studied in different in vitro models and is well documented. Forester and Lambert [41] observed inhibition of DPPH radical synthesis by green tea extract by 88–92 %, regardless of the used extraction medium (water, ethanol, ether). Other authors measured the FRAP level in different types of tea (green, black, oolong), and suggested that green tea has the greatest antioxidant potential (one cup has an antioxidative potential equivalent to 100–200 mg of vitamin C) [42]. In in vivo studies green tea extract was shown to have a high ability to inhibit the formation of AOPP (by 90 %). These studies also showed its inhibitory effect on the glycation process, though the authors indicate the need for further studies [43, 44]. However, other researchers observed that green tea extract has no significant effect on the process of glycation [45], whereas Babu et al. [46] noted a decrease in fluorescence of AGE by 50 % in a rat model as well as antioxidant activity and an ability to chelate divalent metal ions. To our knowledge, there have been no scientific studies evaluating in vitro the inhibitory action of green tea on the formation of high- and low-molecular-weight fractions of AGE and AOPP. Our data show its ability to inhibit T-AGE and T-AOPP formation in a concentration of 10 mM by 52.3 and 69.3 % respectively. In our research inhibition of HMW- and LMW-AGE and HMW-AOPP and LMW-AOPP formation by green tea was between 55.3 and 67.9 %.

Padma 28 and Padma Circosan are herbal multicompound medicines and exhibit a broad spectrum of actions, e.g. a protective effect on blood vessels (both in micro- and macrovascular areas), efficacy in peripheral atherosclerosis, and immunostimulatory and anti-inflammatory mechanisms of action. Padma formulations have also been shown to be able to scavenge free radicals and reduce the oxidation of lipids [17, 19, 26, 47]. In our earlier study [17] we demonstrated their strong ability to inhibit T-AGE and T-AOPP formation (by 56 and 66 %, respectively, for oxidation and glycation). Both Padma preparations in this study also show strong inhibitory action towards T-AGE and T-AOPP formation (55.9 and 64.7 % respectively for Padma 28 and 52.6 and 68.5 % for Padma Circosan). They also had a high inhibitory effect on HMW- and LMW-AGE and HMW-AOPP formation within the range of 49.9 to 74.9 %. In the case of LMW-AOPP they exhibited the strongest action – they show 65.4 and 61.5 % inhibition respectively. Low concentrations of the LMW fractions of AGE and AOPP are highly desirable because they are especially noxious for the human organs and are particularly nephrotoxic. Although the exact mechanism of action LMW is not fully known, some studies have found increased LMW-AOPP levels in patients with kidney disease and also increased occurrence in patients with diabetes. LMW-AOPP are therefore suspected to contribute to secondary diabetic micro- and macrovascular damage, especially of the kidneys [3, 22].

## Conclusions

The presented data show that all of the tested natural substances decreased the level of oxidation and glycation processes, which is reflected by lowering on T-AGE and T-AOPP formation and also HMW and LMW fractions formation in different degrees. In view of their role in the pathogenesis of diabetic complications and the medical and socioeconomic importance of these diseases, it is vitally important to find compounds to inhibit formation of the deleterious AGE and AOPP compounds and especially of their LMW fractions. Our results suggest a possible role of natural substances in the treatment and prevention of diabetic vascular late complications, whereby the two herbal multicompound preparations Padma 28 and Padma Circosan influence the formation of the especially dangerous LMW fractions of AGE and AOPP the most. Nevertheless, the scientific data concerning inhibition of HMW- and LMW-AGE and AOPP are very limited, and more studies are needed, including in an in vivo setting. The obtained results are promising, and due to the significant adverse effects of AGE and AOPP in diabetes, the research will be continued in cell lines or animals.

## Abbreviations

A: Aminoguanidine
AGE: Advanced glycation endproducts
AOPP: Advanced oxidation protein products
BSA: Bovine serum albumin
DPPH: Diphenyl-picrylhydrazyl radical
GT: Green tea
HMW-AGE/HMW-AOPP: High-molecular-weight AGE/AOPP
LMW-AGE/LMW-AOPP: Low-molecular-weight AGE/AOPP
P28: Padma 28
PC: Padma Circosan
Q: Quercetin
T-AGE/T-AOPP: Total AGE/AOPP
VC: Vitamin C

## References

[1] Grzebyk E, Piwowar A (2013). Modyfikacje glikooksydacyjne albuminy w badaniach medycznych. Pol Merk Lek.

[2] Kalousová M, Krha J, Zima T (2002). Advanced glycation end-products and advanced oxidation protein products in patients with diabetes mellitus. Physiol Res.

[3] Thomas M, Tsalamandris C, MacIsaac R, Medley T, Kingwell B, Cooper M, Jerums G (2003). Low-molecular weight AGEs are associated with GFR and anemia in patients with 2 diabetes. Kidney Int.

[4] Thornalley PJ (2003). Use of aminoguanidine (Pimagedine) to prevent the formation of advanced glycation endproducts. Arch Biochem Biophys.

[5] Witko-Sarsat V, Friedlander M, Capeillère-Blandin C, Nguyen-Khoa T, Nguyen AT, Zingraff J, Jungers P, Descamps-Latscha B (1996). Advanced oxidation protein products as a novel marker of oxidative stress in uremia. Kid Int.

[6] Vlassara H, Striker GE (2013). Advanced glycation endproducts in diabetes and diabetic complications. Endocrinol Metab Clin North Am.

[7] Vinson JA, Howard TB (1996). Inhibition of protein glycation and advanced glycation end products by ascorbic acid and other vitamins and nutrients. J Nutr Biochem.

[8] Stirban A, Gawlowski T, Roden M (2014). Vascular effects of advanced glycation endproducts: clinical effects and molecular mechanisms. Molec Metab.

[9] Thomas M, Forbes J, MacIsaac R, Jerums G, Cooper M (2005). Low-molecular weight advanced glycation end products: markers of tissue AGE accumulation and more?. Anm N Y Acad Sci.

[10] Witko-Sarsat V, Nguyen-Khoa, Jungers P, Drueke TB, Descamps-Latscha B (1999). Advanced oxidation protein products as a novel molecular basis of oxidative stress in uremia. Nephrol Dial Transplant.

[11] Tarr JM, Kaul K, Chopra M, Kohner EM, Chibber R. Pathophysiology of diabetic retinopathy. ISRN Ophthalmol. 2013:343560. doi:10.1155/2013/343560. 10.1155/2013/343560 PMC3914226 24563789

[12] Bohlender J, Franke S, Stein G, Wolf G (2005). Advanced glycation end products and the kidney. Am J Physiol Renal Physiol.

[13] Henle T, Deppisch R, Beck W, Hergesell O, Mänsch G, Ritz E (1999). Advanced glycated end-products (AGE) during haemodialysis treatment: discrepant result with different methodologies reflecting the heterogeneity of AGEs compounds. Nephrol Dial Transplant.

[14] Piwowar A, Knapik-Kordecka M, Szczecińska J, Warwas M (2008). Plasma glycooxidation protein products in type 2 diabetic patients with nephropathy. Diab Metab Res Rev.

[15] Munesue S, Yamamoto Y, Urushihara R, Inomata K, Saito H, Motoyoshi S, Watanabe T, Yonekurad H, Yamamoto H. Low-molecular weight Fractions of Japanese soy sauce act as a RAGE antagonist via inhibition of RAGE trafficking to lipid rafts. Food Funct. 2013;4:1835–42. DOI:10.1039/C2FO30359. 10.1039/c2fo30359k 24191276

[16] Poulsena MW, Hedegaardb RV, Andersena JM, de Courtend B, Bügela S, Nielsenc J, Skibstedb LH, Dragsted LO (2013). Advanced glycation endproducts in food and their effects on health. Food Chem Tox.

[17] Grzebyk E, Piwowar A (2014). The Tibetan herbal medicines Padma 28 and Padma Circosan inhibit the formation of advanced glycation endproducts (AGE) and advanced oxidation protein products (AOPP) in vitro. BMC Complement Altern Med.

[18] Grzebyk E, Piwowar A (2016). Inhibition of glycoxidative modification of proteins by some substances of natural origin. Herba Pol.

[19] Ginsburg I, Rozenstein-Tsalkovich L, Koren E, Rosenmann H (2011). The herbal preparation Padma 28 protects against neurotoxicity in PC12 cells. Phytother Res.

[20] Münch G, Keis R, Weßels A, Riederer P, Bahner U, Heidland A, Niwa T, Lemke HD, Schinzel R (1997). Determination of advanced glycation end products in serum by fluorescence spectroscopy and competitive ELISA. Eur J Clin Chem Clin Biochem.

[21] Laemmli UK (1970). Cleavage of structural proteins during the assembly of the head of bacteriophage T4. Nature.

[22] Forbes JM, Soldatos G, Thomas MC (2005). Below the radar: advanced glycation end products that detour "around the side". Is HbA1c not an accurate enough predictor of long term progression and glycaemic control in diabetes?. Clin Biochem Rev.

[23] Chuyen NV (2005). Toxicity of the AGEs generated from the Maillard reaction: on the relationship of food-AGEs and biological-AGEs. Mol Nutr Food Res.

[24] Silva RR, Silva DO, Fontes HR (2013). Alviano CS.

[25] Kerio LC, Wachira FN, Wanyoko JK, Rotich MK (2013). Total polyphenols, catechin profiles and antioxidant activity of tea products from purple leaf coloured tea cultivars. Food Chem.

[26] Colombo G, Clerici M, Giustarini D, Portinaro N, Badalamenti S, Rossi R, Milzani A, Dalle-Donne I (2015). A central role for intermolecular dityrosine cross-linking of fibrinogen in high molecular weight advanced oxidation protein product (AOPP) formation. Biochim Biophys Acta.

[27] Rajasekar P, Anuradha CV (2007). L-Carnitine inhibits protein glycation in vitro and in vivo: evidence for a role in diabetic management. Acta Diabetol.

[28] Losso JN, Bawadi HA, Chintalapati M (2011). Inhibition of the formation of advanced glycation end products by thymoquinone. Food Chem.

[29] Hou FF, Boyce J, Chertow GM, Kay J, Owen WF (1998). Aminoguanidine inhibits advanced glycation end products formation on beta2-microglobulin. J Am Soc Neph.

[30] Gutierrez RMP (2012). Inhibition of advanced glycation end-product formation by origanum majorana L. in vitro and in streptozotocin-induced diabetic rats. Evid Based Complement Alternat Med.

[31] Stadler K, Jenei V, Jakus J (2005). Benefical effects of aminoguanidine on the cardiovascular system of diabetic rats. Diabetes Metab Res Rev.

[32] Ara C, Karabulut AB, Yilmaz M, Kirimliglu V, Yilmaz S (2006). Protective effect of aminoguanidine against oxidative stress in an experimental peritoneal adhesion model in rats. Cell Biochem Funct.

[33] Jing S, Zhang X, Yan LJ (2015). Antioxidant activity, antitumor effect, and antiaging property of proanthocyanidins extracted from kunlun chrysanthemum flowers. Oxid Med Cell Longev.

[34] Shah SM, Ahmad Z, Yaseen M, Shah R, Khan S, Shah SM, Khan B (2015). Phytochemicals, in vitro antioxidant, total phenolic contents and phytotoxic activity of Cornus macrophylla Wall bark collected from the North-West of Pakistan. Pak J Pharm Sci.

[35] Zhang HM, Wang CF, Shen SM, Wang GL, Liu P, Liu ZM, Wang YY, Du SS, Liu ZL, Deng ZW (2012). Antioxidant phenolic compounds from Pu-erh tea. Molecules.

[36] Tarwadi KV, Agte VV (2011). Effect of micronutrients on methylglyoxal-mediated in vitro glycation of albumin. Biol Trace Elem Res.

[37] Lavelli V, Corey M, Kerr W, Vantaggi C (2011). Stability and anti-glycation properties of intermediate moisture apple products fortified with green tea. Food Chem.

[38] Djouossi MG, Tamokou JD, Ngnokam D, Kuiate JR, Tapondjou LA, Harakat D, Voutquenne-Nazabadioko L (2015). Antimicrobial and antioxidant flavonoids from the leaves of Oncoba spinosa Forssk. (Salicaceae). BMC Complement Altern Med.

[39] Wu JW, Hsieh CL, Wang HY, Chen HY (2009). Inhibitory effects of guava (Psidium guajava L.) leaf extracts and its active compounds on the glycation process of protein. Food Chem.

[40] Wu CH, Lin JA, Hsieh WC, Yen GC (2009). Low-density-lipoprotein (LDL)-bound flavonoids increase the resistance of LDL to oxidation and glycation under pathophysiological concentrations of glucose in vitro. J Agric Food Chem.

[41] Forester SC, Lamberd JD (2011). The role of antioxidant versus pro-oxidant effects of green tea polyphenols in cancer prevention. Mol Nutr Food Res.

[42] Benzie I, Szeto YT (1999). Total antioxidant capacity of teas by the ferric reducing/antioxidant power assay. J Agric Food Chem.

[43] Almajano MP, Vila I, Gines S (2011). Neuroprotective effects of white tea against oxidative stress-induced toxicity in striatal cells. Neurotox Res.

[44] Neyestani TR, Shariatzade N, Kalayi A, Gharavi A, Khalaji N, Dadkhah M, Zowghi T, Haidari H, Shab-bidar S (2010). Regular daily intake of black tea improves oxidative stress biomarkers and decreases serum C-reactive protein levels in type 2 diabetic patients. Ann Nutr Metab.

[45] Lunceford N, Gugliucci A (2005). Ilex paraguariensis extracts inhibit AGE formation more efficiently than green tea. Fitoterapia.

[46] Babu PVA, Sabitha KE, Srinivasan P, Shyamaladevi CS (2007). Green tea attenuates diabetes induced Maillard-type fluorescence and collagen cross-linking in the heart of streptozotocin diabetic rats. Pharmacol Res.

[47] Vennos C, Loepfe C (2014). Pathogenese und pleiotrope Behandlungsansätze bei diabetischen Folgeerkrankungen – Übersicht über Wirkmechanismen von Padma 28. Schweiz Z Ganzheitsmed.

